# Computational framework to support integration of biomolecular and clinical data within a translational approach

**DOI:** 10.1186/1471-2105-14-180

**Published:** 2013-06-06

**Authors:** Newton Shydeo Brandão Miyoshi, Daniel Guariz Pinheiro, Wilson Araújo Silva, Joaquim Cezar Felipe

**Affiliations:** 1 Department of Computing and Mathematics, Faculty of Philosophy, Sciences and Languages of Ribeirão Preto, University of São Paulo, São Paulo, Brazil; 2 Department of Biology, Faculty of Philosophy, Sciences and Languages of Ribeirão Preto, University of São Paulo, São Paulo, Brazil; 3 Department of Genetics, Faculty of Medicine of Ribeirão Preto, University of São Paulo, São Paulo, Brazil

## Abstract

**Background:**

The use of the knowledge produced by sciences to promote human health is the main goal of translational medicine. To make it feasible we need computational methods to handle the large amount of information that arises from bench to bedside and to deal with its heterogeneity. A computational challenge that must be faced is to promote the integration of clinical, socio-demographic and biological data. In this effort, ontologies play an essential role as a powerful artifact for knowledge representation. Chado is a modular ontology-oriented database model that gained popularity due to its robustness and flexibility as a generic platform to store biological data; however it lacks supporting representation of clinical and socio-demographic information.

**Results:**

We have implemented an extension of Chado – the Clinical Module - to allow the representation of this kind of information. Our approach consists of a framework for data integration through the use of a common reference ontology. The design of this framework has four levels: data level, to store the data; semantic level, to integrate and standardize the data by the use of ontologies; application level, to manage clinical databases, ontologies and data integration process; and web interface level, to allow interaction between the user and the system. The clinical module was built based on the Entity-Attribute-Value (EAV) model. We also proposed a methodology to migrate data from legacy clinical databases to the integrative framework. A Chado instance was initialized using a relational database management system. The Clinical Module was implemented and the framework was loaded using data from a factual clinical research database. Clinical and demographic data as well as biomaterial data were obtained from patients with tumors of head and neck. We implemented the IPTrans tool that is a complete environment for data migration, which comprises: the construction of a model to describe the legacy clinical data, based on an ontology; the Extraction, Transformation and Load (ETL) process to extract the data from the source clinical database and load it in the Clinical Module of Chado; the development of a web tool and a Bridge Layer to adapt the web tool to Chado, as well as other applications.

**Conclusions:**

Open-source computational solutions currently available for translational science does not have a model to represent biomolecular information and also are not integrated with the existing bioinformatics tools. On the other hand, existing genomic data models do not represent clinical patient data. A framework was developed to support translational research by integrating biomolecular information coming from different “omics” technologies with patient’s clinical and socio-demographic data. This framework should present some features: flexibility, compression and robustness. The experiments accomplished from a use case demonstrated that the proposed system meets requirements of flexibility and robustness, leading to the desired integration. The Clinical Module can be accessed in http://dcm.ffclrp.usp.br/caib/pg=iptrans.

## Background

Translational medicine deals with the application of basic research results, especially those coming from “omics” technologies to help in health and disease processes [1]. This new area of research seeks to reduce the existing gap between the bench and the bedside. This is a big challenge that has many barriers to be overcome and one of the most important is related to the diversity of data. The nature of clinical data is very different from the nature of molecular data, although they are often closely related.

Another significant aspect is that in many cases there is no consensus about what kind of information is most useful and therefore important to relate [2], and there are distinct needs of information for each case, i.e. the kind of information is heterogeneous. Thus, a generic and flexible model tends to be more appropriate. Taking as an example a cancer project that includes several research groups, it is expected that there is a disparity between the databases derived from each project. Thus, to analyze data coming from these sources, it is necessary a platform that processes an effective data integration. In addition, this platform should allow the creation of new types of data easily, to attend the diversity of researchers’ needs.

A global analysis concerning different levels of information is necessary when studying complex mechanisms responsible for the onset of pathological processes. To make it possible, two major aspects of data handling must be well defined and mastered: storage and analysis. It is necessary to provide a computational platform and a data model able to store, represent and integrate clinical and biomolecular information in a consistent way. From a well formalized and structured model it is possible to design consistent methods for computational analysis.

In translational science there are some computational platforms to store and retrieve clinical data. Slim-Prim (Scientific Laboratory Information Management – Patient-care Research Information Management) is an integrated data system for collecting, archiving and distributing basic and clinical research data. Slim-Prim is hosted at the University of Tennessee and provides an open-source version called PRIME [3]. Although Slim-Prim and PRIME claim to allow the management of microarray data information, DNA sequencing information and other biomolecular data, they don’t provide integration to any bioinformatics tools and, at the time, this data are treated like a generic data type.

STRIDE (Stanford Translational Research Integrated Database Environment), developed at Stanford University, is a standard-based platform to support clinical and translational research [4]. It consists of three components: a clinical data warehouse, based on HL7 RIM (Health Level Seven - Reference Information Mode), a semantic model based on ontologies (such as SNOMED, ICD and RxNorm) and a framework to build research management applications. Currently there are no plans to implement STRIDE outside Stanford.

The NIH NCBC (National Institutes of Health - National Center for Biomedical Computing) I2B2 (Informatics for Integrating Biology and the Bedside) is responsible for building applications to manage project-related clinical data in the genomic era [5]. I2B2 Hive is a framework composed of software modules to computationally support clinical research [6]. Each software module is called a “Cell” and each Cell can communicate with each other through Web Services. The main modules are responsible for data storage, ontology management, identity management and others. Although I2B2 Hive is a powerful scalable tool to manage clinical information, it does not have a Cell to represent or to analyze biomolecular data such as microarray or nucleotide sequence data.

In the area of genomics there are several databases of specific organism, disease or biological process and some models of biological databases such as AceDB, Ensembl and Chado that are organism-independent. These specific databases also include analytical tools specific for the problem addressed. CerealsDB [7] is a database of genomic information about wheat. IBDsite [8] is a platform to aggregate and analyze biomolecular data involved in inflammatory bowel diseases (IBD). IPAD [9] and Atlas [10] are more general approaches because they aggregate data from several public genomic databases such as KEGG, GenBank, and Uniprot.

On the other hand, there are biological databases models that are the basis for building computational tools for genomic analysis under an organism-independent way. AceDB (A *C.elegans* Database) [11] is one of the pioneering models for biological databases. It consists of a hierarchical schema of Database Management System (DBMS) and was initially built to support research about *C. elegans* (subsequently adapted to other organisms). It is based on an integrative approach and can be used to represent many other types of information, including those unrelated to biology. Ensembl [12] was initially developed to support human genome research and currently support more than 45 genome species. It consists of several computational tools such as EnsMart [13], a biological data warehousing tool for integration and query of biological data.

A model of biological databases which have gained popularity among research groups devoted to different organisms is Chado [14]. It is a robust, flexible and generic platform that can be adapted to support research related to several organisms. It consists of a modular schema of a relational database that can be adapted and extended. An essential feature of Chado, which differs from the other biological database models is that it is ontology-oriented. Ontologies are structural artifacts used for the representation and integration of knowledge in many domains. Ontologies vary from simple vocabularies, used to standardization of terms, to fully conceptual models that enable reasoning and knowledge discovery. Chado, as well as those other biological databases, does not have a module to store clinical and socio-demographic information.

In this context, we are presenting the definition of a computing framework that aggregates clinical and biomolecular data in a consistent way, allowing the development of computational analysis to be applied in the field of translational medicine. To guarantee standardization and enable further development of generic tools for data analysis we propose the use of a common reference ontology.

We consider to use the Chado model as the basic genomic data model to propose the design and implementation of a new module to store clinical and socio-demographic information, in order to assist procedures and research in translational medicine. Chado was chosen because it is a flexible, robust and ontology-oriented model.

Ontology-anchored approaches have been used successfully to query and integrate data in the clinical and biological domain. CDAO-Store [15] is a computational tool that uses the Comparative Data Analysis Ontology to facilitate the storage and retrieval of phylogenic data. Borlawsky *et al.* reports a proof of concept information retrieval tool called Research-IQ [16], which enables research to query heterogeneous datasets. This approach uses free-text that is mapped to concepts related to osteoarthritis. Payne *et al.*[17] proposes an approach called Constructive Induction to enable the reasoning over a knowledge repository aimed to discover potentially informative biomarker-to-phenotype relationship.

We propose the use of a common reference ontology (Translational Medicine Ontology) to allow data integration through terminology standardization, and to support the development of generic analytical tools. As a use case we have tested our framework to aggregate data from the project “Oncogenomics Applied to Therapy of Head and Neck Carcinoma” sponsored by Brazilian GENOPROT Network (CNPq). Through this framework it is possible to integrate sequence data, gene expression data from microarray, microRNA and disease association data with the clinical and socio-demographic features of patients who provided samples for laboratory test generation.

## Methods

**Figure 1:**
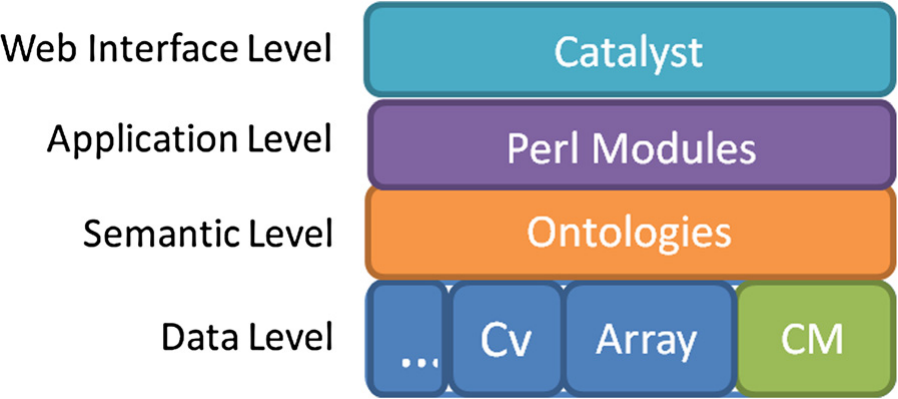
**Levels of the integrative platform for translational research.** In the data level, the green boxes represent the existing Chado modules such as: Cv, controlled vocabulary module and MAGE, microarray gene expression module; the blue box represent the proposed Clinical Module (CM). The semantic level is composed mainly by the clinical database models and the common reference ontology. In the application level there are the Perl modules that implements the business logic. Perl MVC Framework Catalyst implements the user interface level.

The proposed platform is divided in four levels: data level, semantic level, application level and web interface level (Figure 1). In data level we use the Chado model as the basic genomic data model and we have created a new module to store clinical information (Clinical Module - CM). The semantic level consists of the ontologies that represent the clinical databases and a common reference ontology that acts as a conceptual framework. The application level is composed of a set of modules, written in Perl language, that are responsible for the management of the clinical databases, ontologies and data integration process. A web interface allows the interaction between the system and the user. This interface is implemented using the Catalyst Model-View-Controller (MVC) Framework.

We also propose a migration methodology to be applied on legacy clinical databases and an ontological mapping that allows data standardization, integration and development of generic analysis tools.

### Data level

The data level is responsible for storing the data. It is composed of a database management system implementing the Chado data model. We implement the proposed Clinical Module in this level, since Chado has the relations to represent biomolecular data, but does not represent clinical data.

#### Chado

Mungal, Emmert and the Flybase group proposed a modular design based on ontology to represent biological information, called Chado [14]. Chado is a relational database schema that can be used as a basis for any group of genomic research. Chado is part of GMOD (Generic Model Organism Database) project [18] and is currently used by several research groups such as Xenbase [19], ParameciumDB [20], AphidBase [21], BeetleBase [22], among others.

Chado is composed of eighteen modules. Each module is defined as a set of tables, triggers and functions responsible for managing information from a subdomain of genomics. Five out of these modules are the core of Chado. Chado is extensible because it allows the incorporation of new modules and, if necessary, amendments to existing modules.

One hallmark of Chado in relation to other generic databases models is that it makes intensive use of ontologies. Ontology plays a central role in Chado, because all stored information must be related to some ontology or controlled vocabulary. Some ontologies are already incorporated into Chado such as the Sequence Ontology, which is used to describe types of nucleotide sequences and the OBO (Open Biomedical Ontologies) - Relation Ontology, which is used to describe relationships. But it is possible to incorporate new ontologies described in OWL (Web Ontology Language) [23] or in OBO-Format [24].

There are computational tools compatible with Chado databases. These tools are mostly provided by the GMOD group. We can mention the genome browser GBrowse [25] and the Apollo [26] annotation tool. Chado also allows incorporation of other tools through the creation of Bridge Layers which consist of built views to make Chado similar to other databases and act as layers for compatibility with other tools.

#### Proposed clinical model

**Figure 2:**
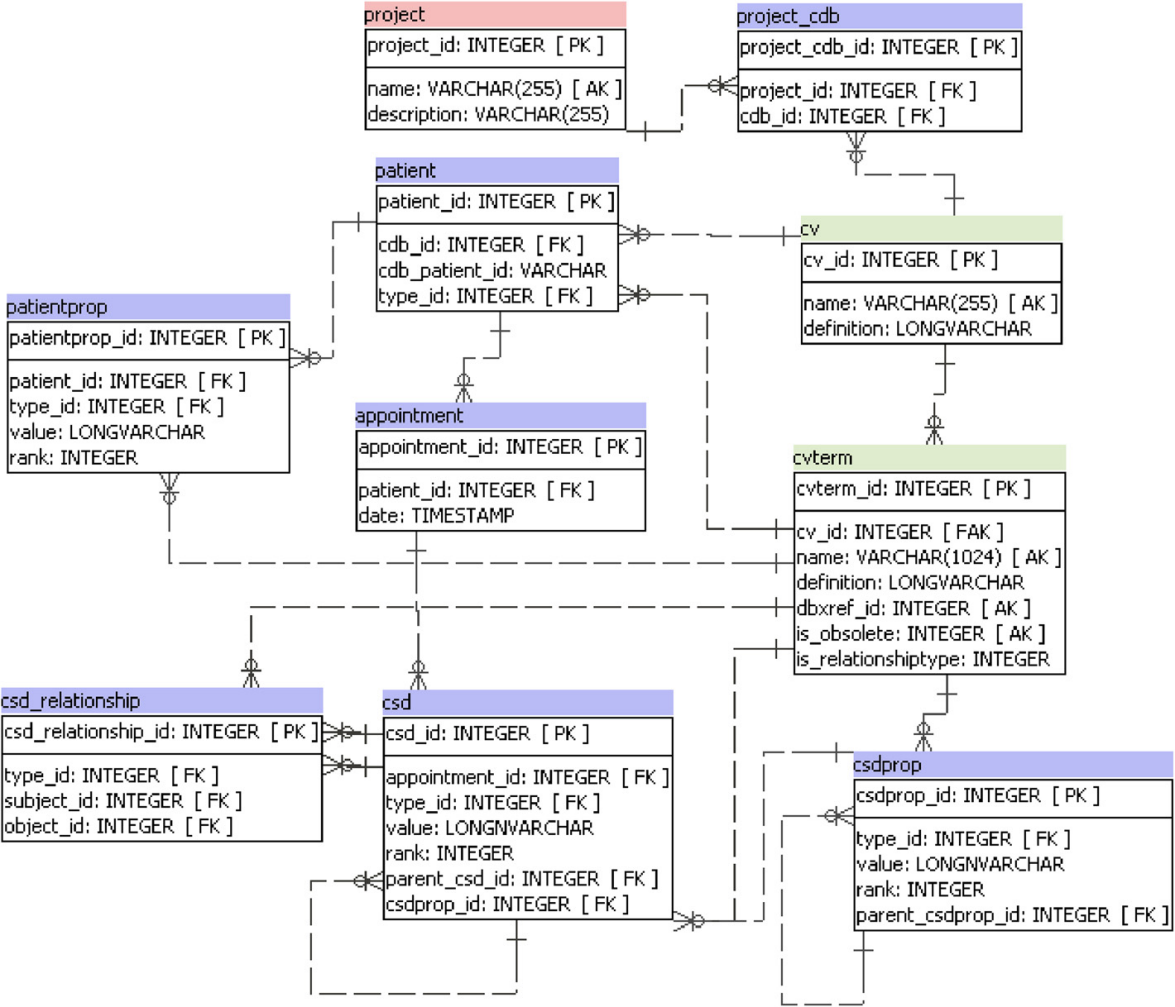
**Chado clinical module.** In green: tables from Controlled Vocabulary module. In pink: tables from the General Module. In blue: tables from the proposed Clinical Module.

Chado has the Stock Module which allows representation of stock collection in a laboratory. This concept of ‘stock’ can be generalized to represent strain, line, biological entities or individual, therefore it could represent patients. The Natural Diversity Module [27] allows representation of experiment data related to a stock, therefore this module allows to represent clinical information as experiments. However, this approach could make it harder the process of clinical data integration from different sources and would preclude the generation and the use of the Clinical Databases bridge layers. Moreover, we think it’s very important to keep a higher level of semantics associated with the tables that are being used. Thereat, and also because of the inherent complexity associated with clinical information, we choose to develop a new Clinical Module. This data model, proposed in this work as a new module of Chado, is shown in Figure 2. An ontology stored in the Controlled Vocabulary (CV) module of Chado defines the semantics of the clinical data stored in this module. This ontology could be any one of those belonging to the biomedical domain, representing the concepts of the clinical data.

The proposed module was designed to be a flexible and generic tool for representation of legacy clinical databases. In Figure 2, the pink table comes from the General Module of Chado, the green tables come from Controlled Vocabulary Module and the blue tables make up the proposed Chado Clinical Module.

The clinical module was built based on the Entity-Attribute-Value (EAV) model. In EAV model the information is represented as a tuple of 3 itens: 1) the *entity,* an identifier of the item or individual which is been described; 2) the *attribute*, the feature described about the item; 3) the *value*, which is the value of that feature applied to the individual. EAV model is best applied mainly when data is sparse, highly heterogeneous, the number of attributes is large and new attributes are often needed. This is the case of clinical data repository or research databases especially those dealing with a large range of medical specialties [28].

The Clinical Module is composed of seven tables: *patient*, *patientprop, appointment, project_cdb*, *csd*, *csd_relationship* and *csdprop*. The *patient* table is self-described, it is where the patients data are stored. Chado already has a *project* table, which defines a context grouping a set of related information such as a set of assays in a study. Since each patient belongs to a clinical database and each clinical database can be linked to many projects, we created the table *project_cdb*.

The clinical or socio-demographic information that do not change over time or that do not require a temporal record (e.g. sex, birthdate, address), are stored in the table *patientprop.*

The types of clinical or socio-demographic information (such as age, weight, tumor size, type of tumor) are represented by an ontology that is stored in the Controlled Vocabulary Module, particularly in *cv* and *cvterm* tables.

The *csd (clinic-social data)* table is where most of the information is stored. This table was designed to represent, in a flexible way, any kind of clinical or socio-demographic information related to a patient. The patient’s information is linked through the *patient_id* column. The semantics of the clinical information is given in column *type_id* that is a foreign key to column *cvterm_id* in *cvterm* table which stores the terms of the ontology that represent the types of clinical and socio-demographic information. The column *value* holds the content of information. The column *rank* is used when it is necessary to store the same type of information to the same patient. For each instance of that information, the column *rank* receives a new value. Another important column is *parent_csd,* which is a self-relationship. This column is used to represent information related to another patient clinical data.

The *csdprop* table is responsible for storing patient-independent information. Usually this kind of data is stored in tables that are referenced by foreign keys in patient table or in any patient-dependent table, for example information about cities, drugs, hospitals, procedures, etc. This kind of information exists regardless of the patients.

The table *csd_relationship* is used when it is necessary to represent complex relationships between clinical or socio-demographic data. In this table it is possible to link two clinical information, using columns *subject_id* and *object_id* which are foreign keys of *csd* table, through a relationship given by the column *type_id,* which is a foreign key of *cvterm* table.

### Semantic level

**Figure 3:**
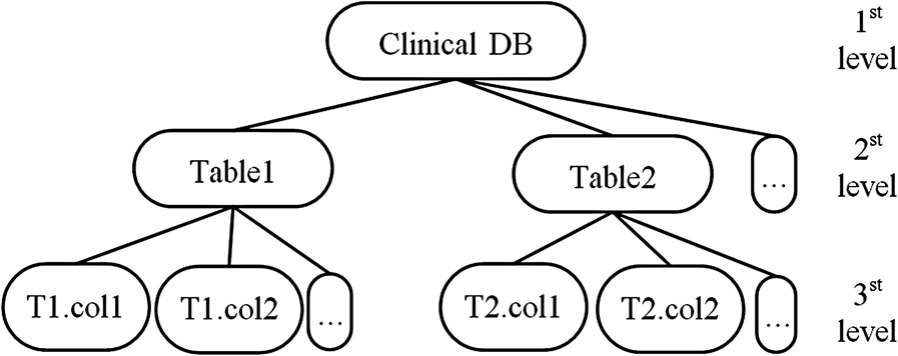
**Clinical database model representation.** Three-level model: first level is a root element representing the clinical database, second level is made up by the tables and third level by the columns.

The Semantic Level is composed of a set of ontologies and database models. According to Rubin et al. [29], ontologies can be a wide variety of computational artifacts such as: terminologies, thesaurus, controlled vocabularies, information models and formal defined ontologies themselves. We can classify the models stored in the semantic level in three different ways:

• Clinical Database Model: these models will describe the structure of a clinical database. In order to do this, it is necessary to represent the tables and their corresponding columns. The model has three levels: the first is the generic element root which, by convention, is the name of the legacy database; the second level is composed of the tables; and in the third level the columns linked with their respective table are represented (Figure 3).

• Domain Ontologies: ontologies that represent concepts from a specific domain of interest, e.g.: ICD, SNOMED, Translational Medicine Ontology and Gene Ontology.

• Common Reference Ontology: it is an ontology used to integrate the clinical information from different Clinical Database Models. It can be composed of one or more domain ontologies. This ontology is used like a conceptual framework where the information is integrated through ontological mappings between concepts of a clinical database model and the common reference ontology.

### Application level

The application level is composed of a set of modules responsible for creating, updating, retrieving and managing information. These modules are written in Perl language to facilitate the integration with tools built to work with Chado.

### User interface level

**Figure 4:**
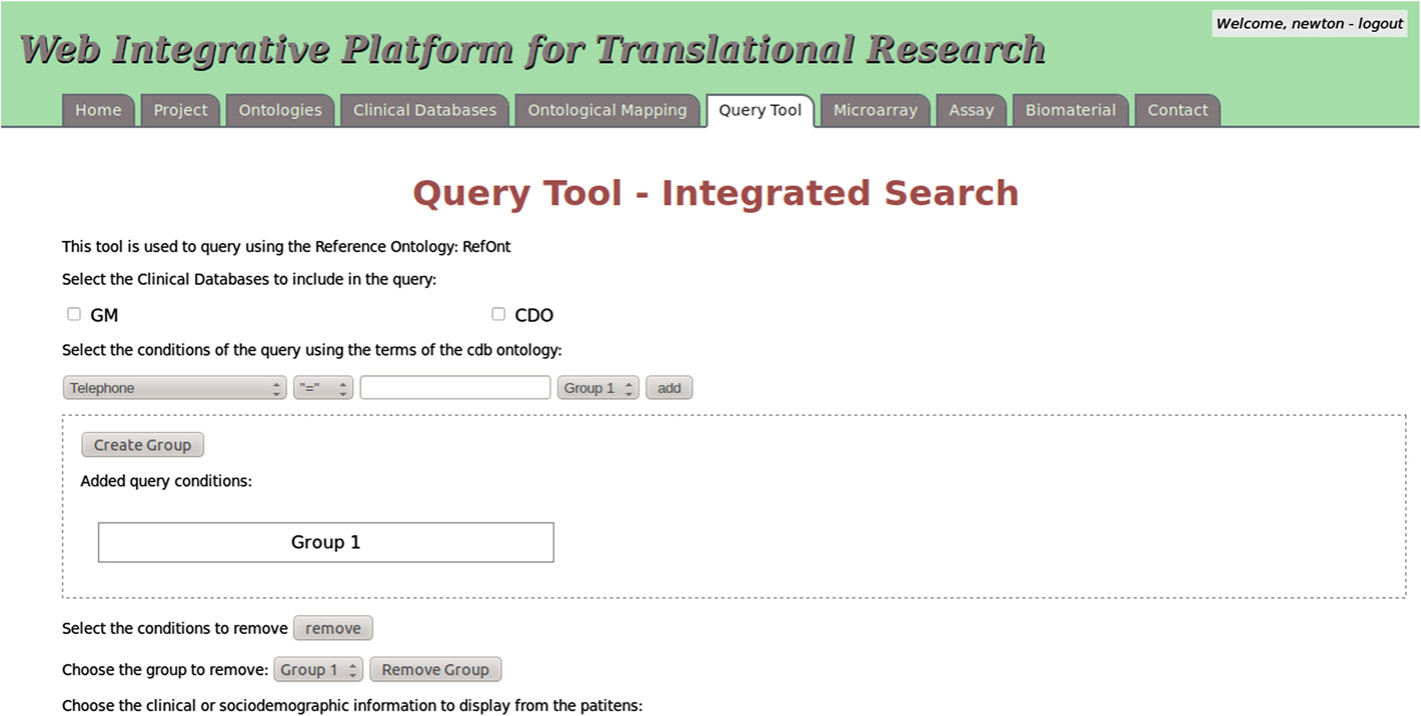
**Web user interface.** Print screen of the query tool interface.

Catalyst is the Perl MVC Framework for building web applications [30]. It is possible to design and implement web application in a modular, maintainable and testable manner. We have used Catalyst to implement the web user interface level. It resulted in a tool called IPTrans (Integrative Platform for Translational Research), whose query interface is shown in Figure 4. Besides supporting the management of clinical and socio-demographic information, this application also supports the management of projects, microarray assays and biomaterials.

### Proposed migration methodology

We have developed a methodology to migrate data from legacy sources to the Clinical Module. The data sources can be of different types, such as relational databases, comma-separated values (CSV) files or SQL dump files.

The methodology consists of four steps:

### Step 1. Create the clinical database model

This step consists of the creation of a model to describe the clinical database (CDB) which houses the original data, as described in the Semantic Level section.

### Step 2. Store the clinical database model on chado

Since the clinical database model is structured in a hierarchical way, it can be represented as a basic ontology. There are several ways to store the created ontology in Chado. It depends on the language used for representation. The most common ontology representation languages are OWL (Web Ontology Language) and OBO-Format (Open Biomedical Ontologies). A simple way is to use the Perl scripts provided with Chado. The clinical database model is represented mainly in the Chado tables *cv* and *cvterm*.

### Step 3. Store the data in the clinical module

In order to migrate the data stored in the legacy clinical database to the Clinical Module in Chado, it is necessary to plan an ETL (Extraction, Transformation and Load) process. In this step, it is important to maintain the correct “typing” information according to the ontology of clinical database stored in the CV module. In other words, it is necessary to correctly relate the information stored with the respective term of the clinical database model.

### Step 4. Create the clinical database bridge layer

The bridge layer consists of a set of views that represent the structure of the clinical research database through Chado. The advantage of creating the Bridge Layer is the facility to query and to adapt the analytical tools that were designed for the clinical database to work correctly on Chado.

The migration methodology proposed here can be used to adapt legacy clinical databases to the proposed framework. This methodology can be applied to data in relational database, comma-separated files and sql dump files. The integration occurs when the ontologies of the clinical sources are mapped to a common reference ontology.

### Ontological mapping

**Figure 5:**
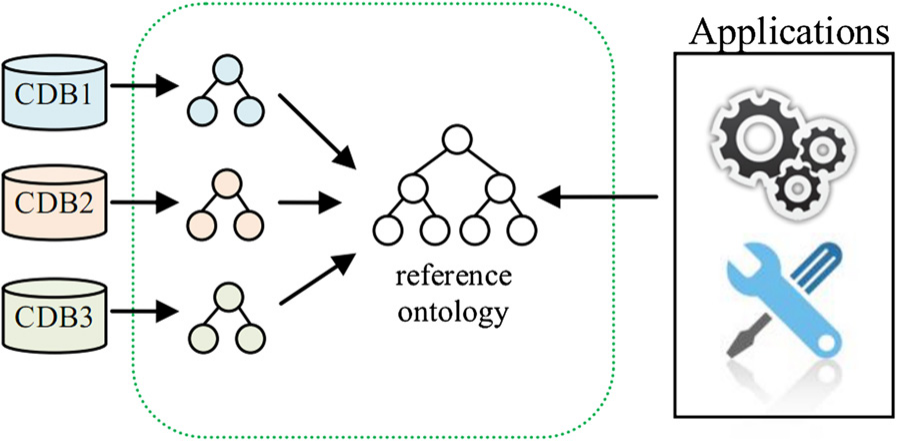
**Ontological mapping between clinical databases models and the reference ontology.** Diagram representing the process of ontological mapping. First the clinical database models are created from the clinical data sources and then each element is mapped to a reference ontology.

The key advantages of the developed platform are the flexibility and the generality to represent information. On the other hand, the proposed structure does not define the meaning of the stored information. The information stored in clinical databases could be represented using specific ontologies that capture the meaning of data in the particular database. But to get the most out of this generic model, allowing the development of analytical tools that could be applied in different instances of Chado with data descending from different clinical databases, it is necessary to define a common semantic. This can be done by adopting a reference ontology, so the analytical tools could be designed to get semantic information from the reference ontology. The work then consists of ontological mapping between the model that describes the clinical database and the reference ontology (Figure 5).

In the following, we formally define the notion of the ontological mapping environment and the rule of the common reference ontology.

**Definition:** An ontological mapping environment is a 5-tuple concept: OME = (CRO, S_k_, SM_*k*_, M_*k,*_ m), k=1..n, where:

• CRO is the common reference ontology, which is a domain ontology that represents general concepts from biomedical domain, acting as a mediated schema. The set of ‘*p*’ concepts and ‘*q*’ relations of CRO are defined as C_1_,…,C_p_ and R_1_,…,R_q_ respectively.

For each k = 1,…n

• S_k_: is a source schema, representing the schema of a clinical source. Examples of clinical sources are tabular files, SQL dump files and relational databases. Each source is composed by a set of ‘*r*’ entities defined as E_1_,…,E_r_.

• SM_k_: is the source model, that uniquely and formally describe S_k_ using a simple hierarchy of terms. SM_k_ is composed by a set of ‘*r*’ terms defined as T_1_,…,T_r_. Each term represents an entity of S_k_.

• M_k:_ is the set of mapping relations that are defined between SM_k_ and CRO. Each mapping relation ‘*m*’ is defined as an *exact match* between a C_i_ and T_i_. M_k_ is composed by ‘*o*’ mapping relations where *o* ≤ *r*.

• M: is defined as the set of all M_k_, in other words, it corresponds to all mapping relations between all SM_k_ and the CRO.

In this work we propose the use of Translational Medicine Ontology (TMO) [31] with mappings to the ACGT (Advance Clinico-Genomics Trials on Cancer) Master Ontology [32] as the common reference ontology.

TMO is an ontology built by the Healthcare and Life Sciences (HCLS) interest group in the W3C Semantic Web. TMO aims to represent general concepts related to translational medicine. It is based on three external ontologies: Basic Formal Ontology, Relation Ontology and Information Artifact Ontology. TMO also have mappings to about other 40 ontologies, e.g. TMO is mapped to the ACGT Master Ontology. The ACGT Master Ontology is an ontology dedicated to cancer research and has been developed in the context of the ACGT project.

The use of TMO with mappings to the ACGT Master Ontology enables the representation of general concepts in the area of translational medicine and it has achieved a greater specificity in the field of oncology allowing representation with greater granularity of information in this area. Through TMO it is possible to extend the platform to other areas of translational medicine using one of the ontologies already mapped to TMO or by extending it.

### Data integration

**Figure 6:**
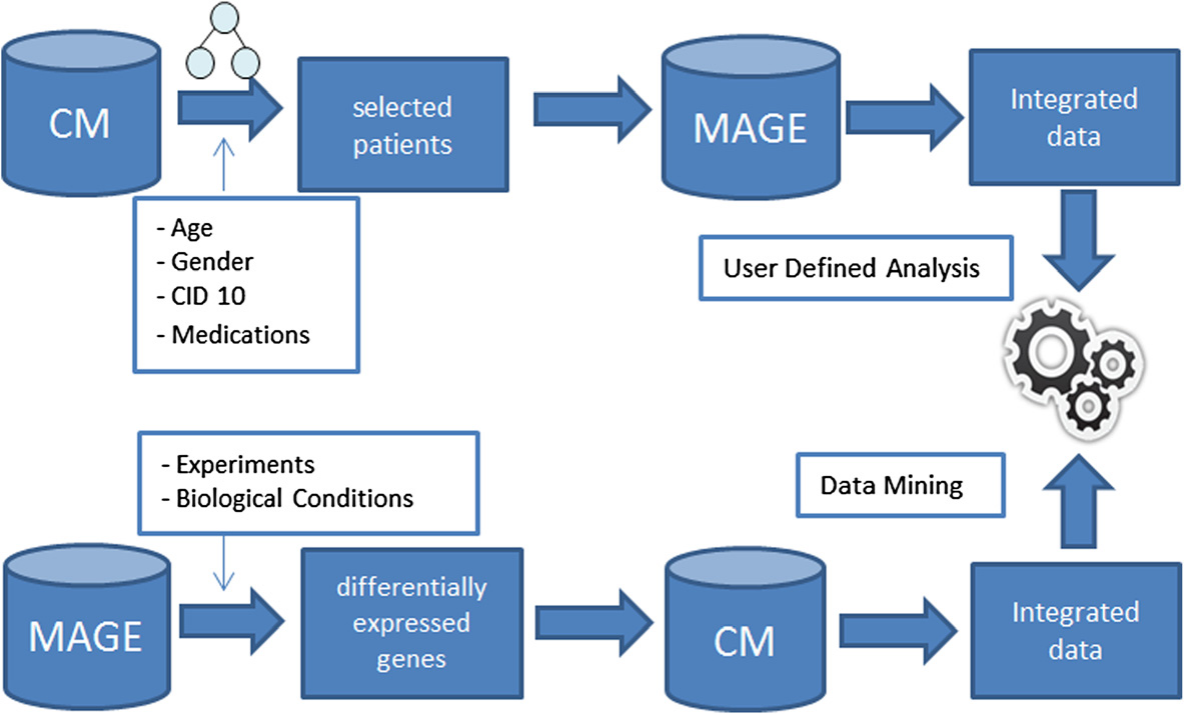
**Example workflow for the generation of integrated data.** Diagram representing two ways of how to generate integrated data. Selecting a group of patients using the reference ontology concepts or selecting a set of experiments using filters with biological conditions.

The user can obtain the integrated data in two different ways through a query tool: the user defines a set of patients based on clinical or socio-demographic characteristics and then the tool outputs the related biomolecular information, such as the gene expression from microarray experiments. The other way is opposite: from a selection of biomolecular information it is possible to obtain the clinical or socio-demographic information related to the set of patients that originated that biomolecular information. This integrated data set can be used in a user-defined analysis or data mining algorithms can be adapted to search for associations between the biomolecular and clinical information (Figure 6).

The integration between clinical information and gene expression information in Chado occurs by the link between the tables *patient* and *biomaterial*. The *biomaterial* table is part of the MAGE Module and is responsible for representing some biological material such as tissue, cells and serum. The patient identification is stored as a property of the biological material in the *biomaterialprop* table. The *assay* table represents a hybridization. The link between the biomaterial and the expression information occurs in *assay_biomaterial* table which maps each biomaterial that is used in each hybridization. In that way, it is possible to associate expression information with clinical or socio-demographic patient data.

## Results

### Use case

To test the proposed framework we have implemented an instance of Chado using the DataBase Management System PostgreSQL 8.4 [33]. We also have implemented the proposed Clinical Module.

We have tested the functionality of this approach with success with data from the project “Oncogenomics Applied to Therapy of Head and Neck Carcinoma” from GENOPROT Network (CNPq - Brazil), whose information is stored in the database of Clinical Genomics Project, which is part of the Ludwig/FAPESP Human Cancer Genome Project. This project aims to carry out joint research focused on the analysis of genetic and epigenetic mechanisms responsible for regulating the transcriptome and secretome in head and neck carcinomas. This research focus on searching for biomarkers for diagnosis and prognosis to allow the use of them as therapeutic targets.

Clinical and demographic data were obtained from patients with tumors of head and neck through the Service of Head and Neck Surgery in School Hospital of Faculty of Medicine (SH-FM) of University of São Paulo, at Ribeirão Preto, Brazil. These patients provided the biomaterial for the assays.

A Chado instance was installed on the relational DBMS PostgresSQL. The clinical database has about 20 tables with some of them containing up to 120 columns. The main table stores information about the patient like age, sex, weight and height. The legacy clinical information was stored in a MySQL [34] relational DBMS.

To implement the first step, by building the clinical database model, based on an ontology, we have used the ontology editor OBO-Edit [35] with the knowledge representation language OBO-Format.

During step 2, we loaded the ontology using Perl scripts provided with Chado.

**Figure 7:**
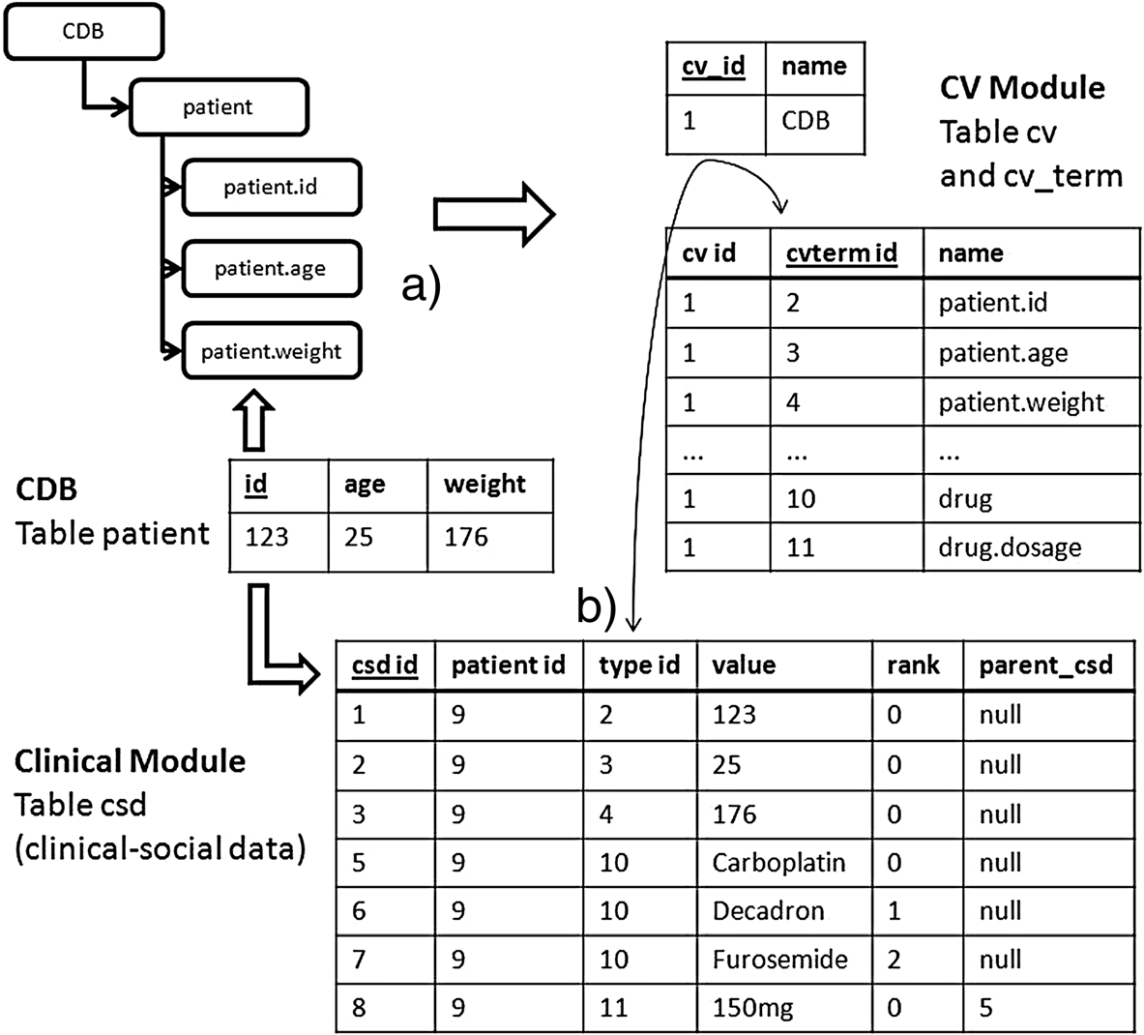
**Integrating the model and the content of a clinical database into Chado CV and clinical module.** Clinical database representation and integration workflow: **a**) Clinical database models are generated and loaded into Chado Controlled Vocabulary Module; **b**) Data are imported into Clinical Module, mainly in table *csd* which follows the EAV structure of information representation.

Figure 7-a shows part of the structure of the patient table in clinical database (CDB) and how this information was stored in the Chado CV module after the ontology loading. The first step consists in representing the patient table and the respective columns such as ‘age’, ‘height’ and ‘weight’ by an ontology and then load this ontology in Chado CV Module. This is done specifically in *cv* table, where the ontologies are stored, and in *cv_term* table, where the ontology terms are stored.

In step 3, we have built an ETL process, which consisted in: extracting the data from the clinical database; transforming the information provided (when necessary); and loading this data in the Clinical Module of Chado. This process was built through the definition of a set of functions in PL/pgSQL. Figure 7-b illustrates how the original information from clinical database can be stored in Chado. In this example, the data extracted from a specific patient are *age, height* and *weight*. First, a record in the *patient* table of Chado Clinical Module is created, and this record receives an internal identifier (in this example, it would be the id “9”). Then, the clinical data and demographic data from this patient as well as the clinical database identifier are stored in the table *csd* of the Clinical Module. This information will be differentiated from each other through the column *type_id,* that is foreign key of column *cvterm_id* from table *cv_term,* which stores the clinical databases ontology. Each piece of information stored in the table *csd* is “typed”, in other words, is semantically represented by the clinical database model stored in clinical CV Module.

To represent part of the information from the clinical database we used the columns *rank* and *parent_csd*. The column rank received a serial value and was used when we stored the same type of information (same *type_id*) for the same patient. A sample case is when we wanted to store the drugs taken by a patient (suppose Carboplatin, Decadron and Furosemide). In this case, the tool automatically created three records in the table *csd* for the same patient (same *patient_id*) and with the same *type_id* (*cvterm* referenced the term "drug"), so each drug is distinguished by a different value of rank.

The column *parent_csd* represents a self-relationship. It was used to store the dosage of the drugs, suppose Carboplatin, one of the drugs that were mentioned in the previous paragraph. To relate the information of dosage to the right drug, we used the column *parent_csd*. Figure 7-b also illustrates the structure and content of the table *csd* for the discussed example.

**Figure 8:**
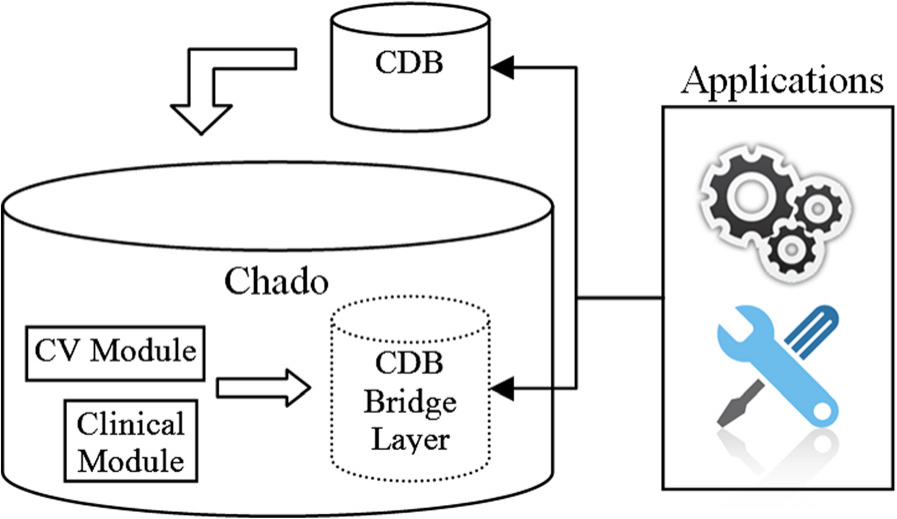
**Clinical database bridge layer.** Clinical database Bridge Layers are generated from clinical database models stored in CV module and represent the original structure of the sources which are used to adapt legacy applications.

Finally, a Bridge Layer for the clinical database in Chado was built, together with a web tool to run on the CDB information management database. The Bridge Layer adapts the web tool to Chado, as well as other applications (Figure 8).

The Bridge Layer could be built for other databases through a portion or all information stored in the clinical module. Thus, other tools and applications built for other databases could be used without recoding.

### Limitations and future work

The proposed computational platform, yet flexible and generic has some limitations: the clinical module is not the best choice to implement hospital common daily procedures such as hospital bed control, drug control, billing and scheduling appointments. The flexibility and generality of the clinical module, which are important in the process of data integration, make the implementation of these functionalities complex and costly. Another issue is the loss of performance in queries over the bridge layer. This happens because the views that compound the bridge layer are built through pivoting the table that follows the EAV model, *i.e.*, the transformation of row modeled data to column modeled. One way to solve this is the materialization of the views that make up the bridge layer. In this solution the data become redundant but there is a performance gain that is higher as the number of columns that compound the view. This same problem occurs in the *ad hoc* query tool. In this case the materialization is not a good strategy because the queries vary widely. Then, we applied the solution proposed by Dinu and Nadkami [28] which consists of breaking a big query into smaller and simple queries and accomplish the union or intersection of the results.

Much remains to be done to meet the computing needs of translational research. Future work can be divided into two contexts: data integration and clinical issues.

In the context of data integration, implementation of entity resolution algorithms would allow identification of the same entity in different databases that are integrated. Schema matching algorithms could be applied to guide the process of mapping between the clinical database models and the reference ontology. One possible solution would be to use the platform OpenII [36]. It would be possible to integrate the OpenII tool into IPTrans data integration methodology.

In terms of clinical information, it would be important to extend the Clinical Module to allow importation of data that follow health information standards like HL7. It would also be important to provide support to DICOM medical image standard. Another aspect would be a security module to implement anonymization algorithms.

## Conclusions

Turning knowledge generated by sciences in a real benefit to enhance human health is one of main goals of translational research in medicine. To make this real, a computing infrastructure is required to support storage, management, integration and analysis of both biological and clinical information.

The presented approach aims to take a step toward this infrastructure, proposing a computational platform that enables the representation of clinical, socio-demographic and biological information in a integrative database, supported by an ontological environment in a flexible and robust way. This platform was designed with a four level architecture: data level, semantic level, application level and user interface level.

Chado biodatabase model was extended to include a module for representing clinical information. Through the proposed clinical information module different clinical databases can be adapted and integrated. The real benefit of adopting a generic model for information representation becomes concrete with the emergence of various applications and analysis tools that are constructed and maintained by the community that adopts this model. It also facilitates the integration of applications and the exchange of data between research groups and also for research groups that do not adopt Chado and may wish to use it after the proposed extension.

The adoption of Chado as the basic model of biological database allows the reuse of the existing tools built from Chado or adapted to it through bridge layers for analysis and visualization of molecular data. With the proposal of the Clinical Module, this solution becomes a robust way to practice translational medicine.

By the use of an ontological approach, through building the semantic level, it is possible to manage and integrate highly heterogeneous data types such as the clinical and socio-demographic data. The common reference ontology acts as a conceptual framework, enabling the mapping of clinical information from different sources to a unique reference.

The practical use of this platform with the real use case demonstrated the feasibility of the integration proposal, highlighting its characteristics of flexibility and robustness.

Through this computational framework we are giving a new step to fulfill the technological gap that exists between the bench and bedside, allowing the reuse of bioinformatics tools and also enabling a flexible way to integrate different sources of clinical and socio-demographic information.

### Availability

The Clinical Module and the instructions to import this approach can be obtained in http://dcm.ffclrp.usp.br/caib/pg=iptrans. It is recommended to use a fresh install of Chado model in a PostgreSQL relational database.

## Competing interests

The authors declare that they have no competing interests.

## Authors’ contributions

JCF and WASJ conceived the project. NSBM made the platform design and implementation. JCF coordinated the project and supervised the design and implementation processes. WASJ provided the access to the clinical data. DGP participated in the evaluation and selection of the data model. All authors helped to draft the manuscript. All authors read and approved the final manuscript.
